# 3D imaging‐based AI models outperform demographic models and excel in tibial sizing compared with 2D models in total knee arthroplasty planning: A systematic review

**DOI:** 10.1002/ksa.70262

**Published:** 2026-01-08

**Authors:** Randa Elsheikh, Zainab Aqeel Khan, George Mihai Avram, Rolf Huegli, Andrej M. Nowakowski, Michael T. Hirschmann

**Affiliations:** ^1^ University Department of Orthopedic Surgery and Traumatology Kantonsspital Baselland Bruderholz Switzerland; ^2^ Department of Clinical Research, Research Group Michael T. Hirschmann, Regenerative Medicine & Biomechanics University of Basel Basel Switzerland; ^3^ Karolinska Institute Stockholm Sweden; ^4^ Institute of Radiology and Nuclear Medicine, Kantonsspital Baselland Bruderholz Switzerland

**Keywords:** artificial intelligence, implant sizing, machine learning, preoperative planning, total knee arthroplasty

## Abstract

**Purpose:**

Accurate preoperative implant sizing is a critical component of successful total knee arthroplasty (TKA). Artificial intelligence (AI) has emerged as a promising tool for enhancing preoperative planning. This is achieved through predictive modelling based on different input modalities, including computed tomography (CT), plain radiographs and patient demographic data. Despite growing interest, the comparative performance of these models remains unclear. This systematic review aims to evaluate and compare the predictive accuracy of AI‐based models for TKA component sizing across different input modalities.

**Methods:**

A systematic literature search was conducted in PubMed, Scopus, Embase and Cochrane Central following the Preferred Reporting Items for Systematic Reviews and Meta‐Analyses guidelines. Eligible studies included original research that developed or validated AI models for predicting component sizes in TKA planning and reported measurable performance outcomes. Methodological quality and risk of bias were assessed using the PROBAST tool. Qualitative synthesis was performed with stratification by planning modality.

**Results:**

Thirteen studies encompassing 37,002 patients met the inclusion criteria. AI models were developed using three‐dimensional (3D) imaging (*n* = 4), two‐dimensional (2D) radiographs (*n* = 4), demographic data (*n* = 2) or mixed inputs (*n* = 2). For femoral component prediction, the highest exact size accuracy was achieved by x‐ray‐based models (86.70%), followed by mixed‐input (86.27%), CT/MRI‐based (79.98%) and demographic models (45.72%). Accuracy within ±1 size remained high across modalities: CT/MRI (97.67%), x‐ray (96.35%) and demographic (92.35%). For tibial components, exact size prediction was similarly high in mixed‐input (85.29%), CT/MRI‐based (83.98%) and x‐ray‐based models (83.57%), while demographic models lagged (52.25%). Prediction within ±1 size exceeded 94% for all modalities, with CT‐based models achieving the highest accuracy (98.49%).

**Conclusion:**

AI models using 2D and 3D imaging achieve high accuracy in TKA component sizing, with 3D imaging performing best for tibial components. Demographic‐only models are less accurate, whereas multimodal approaches may optimize predictive precision in surgical planning.

**Level of Evidence:**

Level III.

**Abbreviations**
2D: two‐dimensional
3D: three‐dimensional
AI: artificial intelligence
AUC: area under the curve
BMI: body mass index
CT: computed tomography
DL: deep learning
MAE: mean absolute error
ML: machine learning
MRI: magnetic resonance imaging
PSI: patient‐specific instrumentation
RMSE: root‐mean‐squared error
TKA: total knee arthroplasty

## INTRODUCTION

Despite the pain and function, long‐term outcomes are closely linked to the precision of component positioning and sizing, with inaccurate sizing often being associated with reduced patient satisfaction and suboptimal joint kinematics, often leading to early revision [2, 44, 46].

To anticipate final component size, plan bone resections and assess alignment strategies, preoperative templating has traditionally been used by surgeons in TKA [25]. Currently, two‐dimensional (2D) radiographic templating using plain anteroposterior and lateral radiographs remains the most commonly used technique owing to its accessibility and low cost [38]. However, this approach is inherently limited by its inability to fully capture the three‐dimensional (3D) anatomy of the knee and is subject to magnification errors and operator‐dependent variability [41].

To improve accuracy, modern planning techniques such as patient‐specific instrumentation (PSI) have been introduced. These systems use morphometric data derived from preoperative computed tomography (CT) scans to generate a personalized surgical plan [45]. Despite the theoretical advantages of PSI, studies have reported discordance between predicted and implanted component sizes. Digital templating using plain radiographs has been shown to result in templating‐implant mismatches in up to 48% of femoral and 55% of tibial components [1, 47]. Even with PSI platforms, disagreement rates of up to 51.1% between the manufacturer's plan and the final implant size, and 26.6% between the manufacturer's and surgeon's plan, have been reported [8]. These mismatches may lead to inefficient instrument tray usage, increased operative time and higher costs, particularly in settings with limited implant inventory [7].

Artificial intelligence (AI) has emerged as a promising solution to the limitations of traditional templating. With applications spanning image segmentation, anatomical landmark detection and predictive analytics, AI has demonstrated strong potential in enhancing surgical planning [3]. In the setting of TKA, AI models have been developed to predict optimal implant sizes based on diverse planning modalities, including demographic features [20], 2D radiographic measurements [5] and 3D imaging data such as CT and magnetic resonance imaging [9, 24]. These models utilize machine learning (ML) and deep learning (DL) models to capture complex, non‐linear relationships between input features and implant sizing decisions [13, 21, 32, 33, 34, 42].

While individual studies have reported encouraging results [5, 17, 22, 24], a systematic synthesis of the predictive performance and clinical applicability of AI‐based implant sizing models in TKA is lacking. Importantly, it remains unclear how planning modality influences predictive accuracy and whether the added complexity of the computational demands of 3D imaging‐based models yields clinically meaningful improvements.

The aim of this systematic review is to evaluate and compare the performance of AI‐based models for preoperative implant size prediction in TKA. Specifically, model accuracy in predicting exact, ±1 and ±2 size matches for femoral and tibial components across different input modalities is assessed. Secondary objectives include the evaluation of computational efficiency, error metrics and comparison with human‐level predictions. It was hypothesized that AI models trained on 3D imaging data demonstrate superior predictive accuracy across all thresholds compared to those based on 2D radiographic or demographic data.

## MATERIALS AND METHODS

### Search strategy and eligibility criteria

This systematic review was conducted in alignment with the Preferred Reporting Items for Systematic Reviews and Meta‐Analyses 2020 guidelines [35] and was prospectively registered on the PROSPERO database (registration number: CRD420251007783). A structured literature search was conducted across four electronic databases: PubMed, Embase, Scopus and Cochrane Central Register from database inception to March 2025, with the prospective inclusion of in‐press online publications.

The search strategy combined terms related to AI (including ‘artificial intelligence’, ‘AI’, ‘machine learning’, ‘ML’, ‘deep learning’, ‘DL’, ‘neural network’ and ‘computational’) with terms related to knee arthroplasty (‘total knee arthroplasty’, ‘TKA’, ‘total knee replacement’, ‘TKR’, ‘total joint arthroplasty’ and ‘total joint replacement’) and planning‐specific keywords (‘planning’, ‘component size’, ‘implant size’, ‘implant sizing’, ‘component sizing’, ‘templating’ and ‘size’). The full applied search strategy is provided in Table S1.

Studies were included if they were original articles that developed or validated AI models for preoperative implant size prediction in TKA and reported measurable performance outcomes. Exclusion criteria included studies not conducted on human subjects, lack of explicit reference to TKA, no report of model performance metrics and non‐original studies like reviews, conference abstracts, book chapters or editorials.

Following duplicates removal, title and abstract screening, and full‐text screening were conducted by two independent reviewers using Covidence systematic review software (Veritas Health Innovation Ltd.). Any discrepancy during the screening process was resolved by consensus.

### Data extraction

Data extraction was performed independently by two reviewers using a pre‐standardized extraction form. Disagreements were resolved by discussion. Extracted information included study design, country of origin, total sample size, patient age, sex distribution, body mass index (BMI) and laterality of the assessed knee.

Model‐related data included the source of the trained data, AI methodology (e.g., ML, DL or CNN), algorithm type, primary planning modality (e.g., CT, radiography or demographic‐based) and validation approach (internal or external). Additional variables included segmentation technique, anatomical landmark identification method, device or implant specificity, and whether the model had been clinically integrated. Key extracted outcome measures included model accuracy for femoral and tibial implant sizing. When reported, computational metrics such as average segmentation time, AI‐based component prediction time, patient‐specific implant (PSI) design time and total time from imaging to PSI printing were extracted.

Missing or non‐numeric data were treated as unavailable and excluded from numerical analyses.

### Outcome measures

The primary outcomes of interest were the predictive accuracy of AI‐based models in estimating femoral and tibial implant sizes for preoperative TKA planning. Accuracy was assessed at three thresholds for each component: exact size match, prediction within ±1 size, and within ±2 sizes. Secondary performance metrics included the area under the receiver operating characteristic curve (AUC), mean absolute error (MAE), root‐mean‐squared error [16] and variant RMSE for predictions within ±1 size. When available, models were also evaluated for overhang/underhang prediction accuracy, including maximum correct prediction and prediction within ±1 size of correct over‐ or under‐sizing.

Ground truth determination methods were recorded to assess the reference standard against which model performance was evaluated. Additionally, comparisons with human‐level predictions were extracted when reported.

For analytical clarity, outcomes were stratified by the model's primary input modality: 3D imaging‐based models (CT or MRI), 2D radiography‐based models and models relying on demographic or clinical data alone.

### Study quality, bias and characteristics

The methodological quality and risk of bias for each included study were assessed using the Prediction model Risk Of Bias ASsesment Tool (PROBAST) [49]. PROBAST is a validated framework specifically designed to assess the quality of studies that develop, validate or update multivariable prediction models. Four key domains are assessed: participants, predictors, outcomes and analysis. Each domain contains specific signalling questions that guide the assessment of whether the study design, data collection and analytical methods could introduce systematic bias into the model's development and validation. Based on the responses, each domain is rated as having low, high or unclear risk of bias.

### Statistical analysis and data synthesis

Data synthesis was performed using descriptive statistical methods. Continuous variables were summarized using means and standard deviations, and categorical variables were described using frequencies and percentages. Data entries labelled as not reported or expressed in non‐numeric terms were excluded from quantitative synthesis.

Due to heterogeneity in model architectures, data modalities and reporting practices, formal meta‐analysis could not be performed. Instead, a structured qualitative synthesis was undertaken, with subgroup comparisons based on the planning approach. Computational performance metrics and model accuracies were compared across modalities where sufficient data were available.

All results were presented in tabular format to ensure clarity and facilitate cross‐study comparison. Data processing and visualization were conducted using Microsoft Excel (version 16.54, Microsoft Corporation).

## RESULTS

### Study characteristics

**Figure 1:**
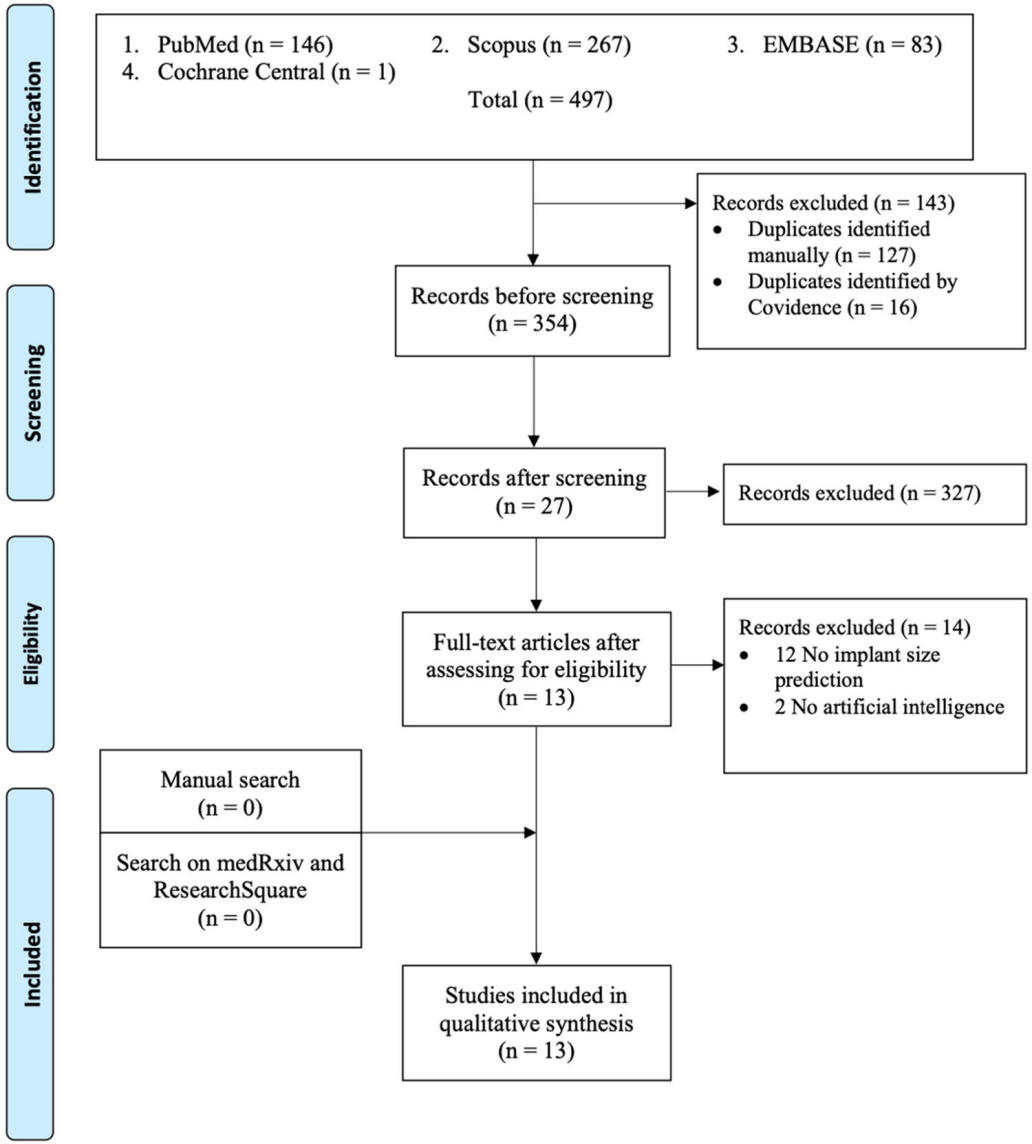
PRISMA flow diagram showing the study selection process. PRISMA, Preferred Reporting Items for Systematic Reviews and Meta‐Analyses.

Of the 497 screened articles, a total of 13 studies met the eligibility criteria and were included in the final analysis (Figure 1). Based on the primary input modality used for model development, four studies utilized 3D imaging (CT or MRI) [17, 22, 23, 27], four relied on 2D radiographs [5, 36, 37, 50], two used demographic data [19, 20] and two combined demographic and radiographic data (mixed models) [51, 52].

The total number of patients used for model training and validation across all studies was 37,002. The average age across the entire data set was 67.98 ± 3.04 years, and the pooled average BMI was 28.00 ± 2.93 kg/m^2^. Sex distribution was reported in most studies, with 17,138 females (46.31%) and 12,972 males (35.05%). Regarding laterality of the training data source, 355 (0.95%) left and 341 (0.92%) right knees were assessed. Further details about the included studies are provided in Table 1.

**Table 1:** Baseline characteristics of the included studies.

Author, year	Country	Study design	Level of evidence	Sample size	Male	Female	Average age (years)	Body mass index (kg/m^2^)
Yue, 2019 [52]	China	Retrospective validation	III	308	69 (22.4%)	239 (77.6%)	NR	NR
Kunze, 2021 [19]	United States	Retrospective cohort	III	17,283	7421 (42.9%)	9862 (57.0%)	66.3 ± 9.4	31.9 ± 6.4
Yue, 2022 [51]	China	Retrospective cohort	III	308	68 (22.0%)	240 (77.9%)	NR	NR
Kunze, 2022 [20]	United States	Retrospective cohort	III	11,777	5305 (45.0%)	6472 (55.9%)	66.5 ± 9.5	31.2 ± 5.6
Lambrechts, 2022 [23]	Belgium	Retrospective validation	III	5409	NR	NR	NR	NR
Burge, 2022 [5]	United Kingdom	Retrospective validation	III	78	33 (42.3%)	45 (57.7%)	62.5 ± 3.74	NR
Li, 2023 [27]	China	Retrospective case‐control	III	42	14 (33.3%)	6 (14.2%)	67.95 ± 5.65	25.11 ± 3.53
Lambrechts, 2023 [22]	Belgium	Retrospective cohort	III	446	NR	NR	NR	NR
Lan, 2024 [24]	China	Retrospective cohort	III	30	10 (33.3%)	20 (66.6%)	69.10 ± 5.98	25.63 ± 3.00
Yu, 2024 [50]	South Korea	Retrospective cohort	III	714	NR	NR	NR	NR
Park, 2024 [37]	South Korea	Retrospective validation	III	234	43 (18.3%)	191 (81.6%)	71.5 ± 5.9	NR
Park, 2024 [36]	South Korea	Retrospective cohort	III	81	9 (11.1%)	63 (77.7%)	72.0 ± 7.6	26.16 ± 4.9
Katragadda, 2024 [17]	United Kingdom	Retrospective validation	III	292	NR	NR	NR	NR

### Risk of bias

**Figure 2:**
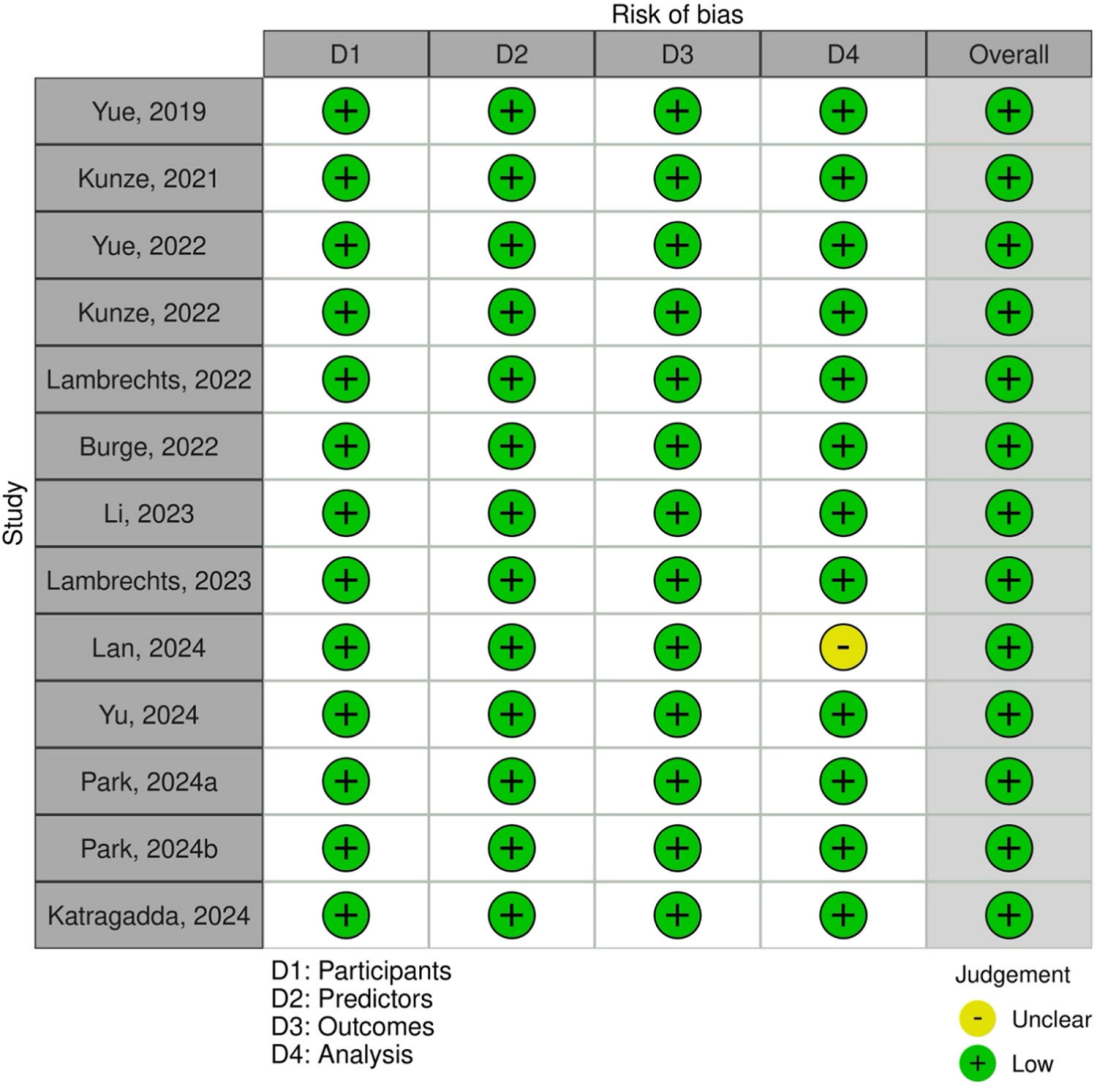
Overall risk of bias in the included studies assessed using the PROBAST tool. Green represents a low risk of bias, and yellow represents unclear risk. PROBAST, Prediction model Risk Of Bias ASsesment Tool.

**Figure 3:**
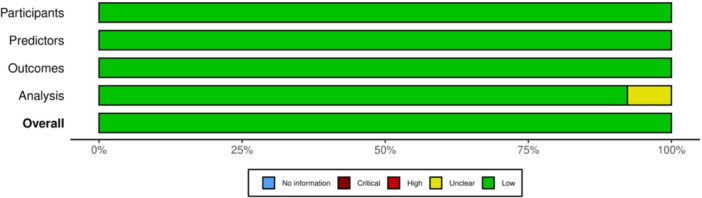
Domain‐specific risk of bias of the included studies assessed using the PROBAST tool. Green represents a low risk of bias, and yellow represents unclear risk. PROBAST, Prediction model Risk Of Bias ASsesment Tool.

Risk of bias was assessed using the PROBAST tool across four domains: participants, predictors, outcomes and analysis. All 13 included studies were rated as low risk in the participants, predictors and outcomes domains. Twelve studies [5, 17, 19, 20, 22, 23, 27, 36, 37, 50, 51, 52] were rated as low risk of bias in the analysis domain, while one study [24] had an unclear risk due to insufficient reporting of statistical methods but was retained given its methodological relevance and contribution to the evidence base. Overall, all included studies were judged to have a low risk of bias (Figures 2 and 3), suggesting a high methodological quality among the assessed studies.

### Model characteristics

Substantial variation in data sources, AI structure, input modalities and clinical applications was present in the included studies. Most models were developed using institutional data sets, incorporating preoperative CT or MRI scans, standard radiographs and patient demographic data.

In terms of model architecture, six studies employed ML models [5, 17, 19, 20, 22, 23], five utilized DL models (primarily CNN‐based) [36, 37, 50, 51, 52] and two described broader AI approaches [24, 27]. Segmentation techniques ranged from fully automated CNN‐based methods to semi‐automated workflows. Landmark identification was achieved via neural networks in most models, though manual annotation was used in some.

Clinical integration varied across studies: several models were intended for PSI, surgical navigation, augmented or robotic‐assisted surgery, or standalone applications for preoperative templating and surgical planning. Similarly, several models were trained on implants from a single manufacturer [17, 20, 22, 24, 36, 37, 50, 51], while others incorporated components from several manufacturers and were, therefore, non‐device specific (Table 2).

**Table 2:** Characteristics of the assessed AI models.

Author, year	Data sources	Model	Algorithm	Planning modality	Segmentation technique	Technique specificity	Implant specificity
Yue, 2019 [52]	Local database	DL	ResNet	X‐ray and demographic data (mixed model)	Contrast‐limited adaptive histogram	NR	No
Kunze, 2021 [19]	Local database	ML	SGB, RF, SVM, XGB, ENPLR	Demographic data	NA	NR	Zimmer Biomet, Corentec, DJ Orthopaedics, Exactech, DePuy Synthes, Stryker, Wright prostheses
Yue, 2022 [51]	Local database	DL	CNN, ECOC, ResNet	X‐ray and demographic data (mixed model)	Contrast‐limited adaptive histogram	NR	Domestic AK posterior stable prostheses
Kunze, 2022 [20]	Local database	ML	SVM, RF, SGB, ENPLR, XGB	Demographic data	NA	NR	Triathlon (Stryker)
Lambrechts, 2022 [23]	Local database	ML	SVR, LAD‐SVR, MTL, Lasso, Group Lasso	CT and MRI	NR	PSI, Navigation, RAS	Vanguard (Zimmer Biomet), Persona (Zimmer Biomet), NexGen (Zimmer Biomet)
Burge, 2022 [5]	OAI and KISTI	ML	U‐Net CNN, PDM, SSM	X‐ray	U‐Net CNN	NR	NexGen (Zimmer Biomet), SIGMA (DePuy Synthes), Legion (Smith & Nephew), Freedom (Maxx Orthopedics), Scorpio (Stryker)
Li, 2023 [27]	Local database	AI	3D U‐Net, HRNet	CT	3D U‐Net, BN, ReLU	PSI	No
Lambrechts, 2023 [22]	Local database	ML	Group Lasso, Elastic Net Linear Regression	MRI	Semi‐automatic segmentation, marching cubes algorithm for surface mesh conversion	NR	PS Vanguard (Zimmer Biomet)
Lan, 2024 [24]	Local database	AI	G‐NET NN	CT	G‐NET NN	NR	DePuy Synthes prostheses
Yu, 2024 [50]	Local database	DL	ResNet, SGD	X‐ray	NR	NR	PS NexGen (Zimmer Biomet)
Park, 2024 [37]	Local database	DL	YOLO, CNN	X‐ray	YOLO	NR	PS and CR Triathlon (Stryker)
Park, 2024 [36]	Local database	DL	CNN, HRNet	X‐ray	CNN	NR	CR Triathlon (Stryker)
Katragadda, 2024 [17]	NR	ML	Linear regression	CT	Active appearance model	Conventional TKA, CAS, RAS, Navigation	PS and CR Triathlon (Stryker)

Abbreviations: AI, artificial intelligence; BN, batch normalization; CAS, computer‐assisted surgery; CNN, convolutional neural network; CR, cruciate‐retaining; DL, deep learning; ECOC, error correct output coding; ENPLR, elastic‐net penalized logistic regression; HRNet, high‐resolution network; KISTI, Korean Institute of Science and Technology Information; LAD‐SVR, least absolute deviation support vector regression; ML, machine learning; MTL, multi‐task lasso; NA, not applicable; NN, neural network; NR, not reported; OAI, Osteoarthritis Initiative; PDM, point depth model; PS, posterior‐stabilized; PSI, patient‐specific instrumentation; RAS, robotic‐assisted surgery; ReLU, rectified linear unit; ResNet, residual network; RF, random forest; SGD, stochastic gradient descent; SGB, stochastic gradient boosting; SSM, statistical shape model; SVM, support vector machine; SVR, support vector regression; TKA, total knee arthroplasty; XGB, extreme gradient boosting; YOLO, You Only Look Once.

Model validation was primarily internal, employing training/validation/test splits, *k*‐fold cross‐validation or retrospective comparisons within the training population. Only one study conducted external validation on a separate data set of patients who had undergone TKA [37].

Models were trained using a variety of imaging and non‐imaging inputs, with demographic and radiographic‐based models relying on a low number of variables and 3D‐based imaging models incorporating substantially higher input dimensionality, with one model using 149 features and another employing 22,476 variables [22, 23]. Output targets also differed by modality. While demographic‐based models generally predicted two output variables, 3D imaging‐based models ranged from predicting a single implant size to up to 14 surgical parameters, including implant dimensions, positioning and alignment metrics. Details about the input and output variables used to train the evaluated models are provided in Table 3.

**Table 3:** Input and output variables of the assessed models.

Author, year	Planning modality	Input variables	Output variables
Yue, 2019 [52]	X‐ray and demographic data (mixed model)	AP and lateral radiographs, sex, height, weight	Exact femoral and tibial component sizes
Kunze, 2021 [19]	Demographic data	Age, height, weight, BMI, sex	Exact, ±1 size, ±2 size femoral and tibial component size
Yue, 2022 [51]	X‐ray and demographic data (mixed model)	AP and lateral radiographs, sex, height, weight	Exact femoral and tibial component size
Kunze, 2022 [20]	Demographic data	Age, height, weight, BMI, sex	Exact, ±1 size, ±2 size femoral and tibial component sizes
Lambrechts, 2022 [23]	CT and MRI	Landmark locations, measurements (femoral notching, mediolateral femoral implant overhang, tibial underhang/overhang), DOFs in MPPs, shape coefficients from SSM	DOFs of the surgeon corrected preoperative plan (including exact femoral and tibial implant sizes)
Burge, 2022 [5]	X‐ray	AP and lateral radiographs	Exact, ±1 size femoral and tibial component sizes
Li, 2023 [27]	CT	Centre of the femoral head, intercondylar fossa, medullary midpoint of the femur and tibia	Exact, ±1 size, ±2 size femoral and tibial component sizes, implant positioning (outlier of LDFA, outlier of MPTA, outlier of HKA, outlier of LDFA ≤ 3°, outlier of MPTA ≤ 3°, outlier of HKA ≤ 3°)
Lambrechts, 2023 [22]	MRI	3D femoral bone mesh vertex coordinates	Exact, ±1 size femoral component size
Lan, 2024 [24]	CT	NR	Exact femoral and tibial component sizes, femoral valgus correction angle, HKA
Yu, 2024 [50]	X‐ray	AP and lateral radiographs	Exact femoral and tibial component sizes
Park, 2024 [37]	X‐ray	AP radiographs	Exact, ±1 size femoral and tibial component sizes
Park, 2024 [36]	X‐ray	AP and lateral radiographs	Exact, ±1 size femoral and tibial component sizes
Katragadda, 2024 [17]	CT	NR	Exact, ±1 size femoral and tibial component sizes

Abbreviations: AP, anteroposterior; DOF, degree of freedom; HKA, hip–knee–ankle angle; LDFA, lateral distal femoral angle; MPP, manufacturer's preoperative plan; MPTA, medial proximal tibial angle; NR, not reported; SSM, statistical shape model.

### Prediction accuracy

Model accuracy varied across planning modalities and implant components (Figure S1). For the femoral component, exact size prediction accuracy was highest for x‐ray‐based models at 86.70%, closely followed by models combining radiographs and demographic data (86.27%) and 3D CT/MRI‐based models (79.98%). Demographic‐only models demonstrated substantially lower exact accuracy at 45.72%. Accuracy within ±1 bucket size remained high across modalities, with CT/MRI achieving 97.67%, x‐ray 96.35% and demographic models 92.35%. ±2 size accuracy reached 100% for CT/MRI models and 99.10% for demographic data. No ±2 size data were reported for mixed or x‐ray‐based models.

For the tibial component, exact size accuracy was comparable between mixed models (85.29%), CT/MRI (83.98%) and x‐ray (83.57%), with demographic data displaying lower prediction accuracy at 52.25%. ±1 size accuracy exceeded 94% for all modalities, with models based on CTs and MRIs displaying the highest prediction accuracy at 98.49%, followed by x‐ray‐based models (96.89%) and demographic‐based models (94.91%). ±2 size tibial accuracy was reported only for models utilizing 3D CT/MRI (100%) and demographic data (99.07%) (Table 4).

**Table 4:** Performance of the assessed AI preoperative planning models classified by the used input modality.

Performance metrics	3D CT/MRI	2D x‐ray	Demographic	Mixed
Accuracy				
Femur				
Exact	79.98%	86.7%	45.72%	86.27%
±1 size	97.67%	96.35%	92.35%	‐
±2 size	100.00%	‐	99.10%	‐
Tibia				
Exact	83.98%	83.57%	52.25%	85.29
±1 size	98.49%	96.89%	94.91%	‐
±2 size	100.00%	‐	99.07%	‐
Error metrics				
Femur				
AUC	‐	0.84	‐	‐
MAE (mm)	0.52	‐	1.68	‐
RMSE (mm)	‐	1.13	2.38	‐
Max over/underhang (%)	‐	71.79%	‐	‐
Tibia				
AUC	‐	0.89	‐	‐
MAE (mm)	0.39	‐	1.68	‐
RMSE (mm)	‐	1.36	2.43	‐
Max over/underhang (%)	‐	72.82%	‐	‐
Computational efficiency				
Time to segmentation (min)	2.49 ± 1.25	NA	NA	NA
Time to component planning (min)	5.98 ± 1.30	0.81 ± 0.03	‐	‐
Time to PSI design (min)	35.10 ± 3.98	‐	‐	‐
Time to PSI printing [4]	19.86 ± 2.44	‐	‐	‐

Abbreviations: AUC, area under the curve; MAE, mean absolute error; NA, not applicable; PSI, patient‐specific instrumentation; RMSE, root‐mean‐squared error.

### Prediction error metrics

Error metrics were primarily available for demographic‐ and CT/MRI‐based models (Table 4). MAE was lowest in 3D imaging‐based models (femur: 0.52 mm; tibia: 0.39 mm) as compared to demographic models (1.68 mm for both components). Root‐mean square error [16] was 2.38 mm (femur) and 2.43 mm (tibia) for demographic data, and substantially lower for x‐ray‐based models (1.13 mm femur, 1.36 mm tibia); RMSE data were not available for CT/MRI‐based models or mixed models. Maximal over‐ or underhang correction accuracy was reported only in one study (femur 71.79%, tibia 72.82%) [5].

### Computational efficiency

Computational efficiency was only reported in three studies [17, 27, 37]. For CT‐based AI models, segmentation required on average 2.49 ± 1.25 min. Component planning times differed substantially, with CT‐based planning taking on average 5.98 ± 1.30 min compared to 0.81 ± 0.03 min for x‐ray‐based models, rendering 2D models planning approximately 7.4 times faster. AI‐based PSI design time in models using CTs took 35.10 ± 3.98 min, with a total processing to printing time of 19.86 ± 2.44 h (Table 4).

### Ground truth determination

Ground truth determination differed across studies depending on the input modality. Among CT/MRI‐based models, one study relied on 3D planning performed jointly by an engineer and surgeon, with independent verification by two blinded physicians using acetate templating on preoperative radiographs [27]. In other studies, ground truth was defined either by the actual prosthesis sizes used intraoperatively, surgeon‐corrected preoperative plans or sizes planned by a single experienced surgeon [17, 22, 23, 24].

In x‐ray‐based models, ground truth definitions included intraoperative sizing trials, actual implant sizes recorded in post‐operative surgical documentation [36, 37, 50], and, in one model, the best‐fit ground truth size was retrospectively calculated based on the lowest error relative to the patient's 3D MRI‐derived bone model [5].

For demographic‐based models, ground truth implant sizes were extracted from automated inventory systems, capturing the final femoral and tibial component sizes implanted during surgery [19, 20].

In mixed models, ground truth was based on surgical records of the actual implanted prosthesis size, as documented in operative reports or inventory systems [51, 52].

### Comparison with human‐level prediction

CT/MRI‐based AI models consistently outperformed manufacturer default plans and manual templating techniques, with several studies reporting significantly higher accuracy and improved outlier rates in alignment metrics [17, 22, 23, 24, 27]. Similarly, x‐ray‐based models achieved higher implant size prediction accuracy than surgeons, showing a stronger correlation with actual implant size [5, 36, 37, 50]. Demographic‐only models were generally less accurate in exact sizing but performed well in ±1 and ±2 size accuracy, approaching or exceeding conventional planning methods in some cases [19, 20]. Mixed‐input models (combining imaging and demographic data) matched or surpassed the accuracy of experienced surgeons and outperformed baseline and traditional planning approaches [51, 52].

## DISCUSSION

The most important findings of this study were that AI‐based models using imaging modalities, particularly plain radiographs and CT/MRI, achieved the highest accuracy in preoperative TKA implant sizing. While x‐ray‐based models showed the highest exact femoral sizing accuracy, CT/MRI‐based models showed superior accuracy within ±1 and ±2 thresholds for both femoral and tibial components. In contrast, demographic‐only models yielded substantially lower exact accuracy (45%–52%), though they maintained acceptable performance within broader sizing margins. Notably, plain radiograph‐based models also demonstrated significantly greater computational efficiency, with planning times up to seven times faster than CT‐based models. Integration of imaging‐based AI planning into clinical practice has the potential to minimize component misalignment and decrease intraoperative adjustments. By reducing operative variability and improving preoperative planning protocols, these models may contribute to improved patient outcomes and more efficient allocation of operative resources.

These findings are consistent with previous research indicating that CT‐based 3D templating methods consistently outperform conventional 2D radiograph‐based methods in preoperative TKA planning [10, 18, 39]. Specifically, studies evaluating 2D digital templating report exact implant size match rates ranging from 34% to 65% for both femoral and tibial components [12, 30, 41], while 3D templating achieves substantially higher accuracy, with exact matches between 80% and 96%, and near‐perfect prediction within one size increment [26, 28, 39].

Demographic data, such as height, weight and sex, have also been explored as predictors of implant size, given the established correlation with femoral component dimensions [43]. However, consistent with the current review, demographic‐only models have shown limited precision, with exact prediction accuracies ranging from 43% to 54% [29, 48]. The lower dimensionality and lack of anatomical context may explain their inferior precision, despite acceptable performance in broader size ranges. Notably, in line with our findings, combining demographic data with plain radiographs has demonstrated improved performance, supporting the potential utility of multimodal input strategies for enhancing preoperative planning accuracy [43].

AI has emerged as a transformative tool in preoperative TKA planning, enabling consistent, data‐driven and personalized surgical strategies [3]. Evidence shows that AI‐based planning models, particularly those utilizing CT imaging, outperform traditional templating in predicting femoral, tibial and liner component sizes, while also reducing operative time by up to 40%, decreasing intraoperative blood loss, improving lower limb alignment and accelerating early post‐operative recovery [11, 31]. Hiraoka et al. further demonstrated that AI‐driven robotic‐assisted workflows, combined with lean principles such as optimizing operating room setup, staffing and protocol standardization, reduced setup time by 4.3 min and minimized instrument set usage, highlighting that AI integration can enhance both surgical precision and overall operational efficiency [14].

In addition to predicting implant size, several AI models have expanded their scope to forecast optimal alignment targets and identify potential implant‐bone mismatches, such as mediolateral overhang, notching or overstuffing [6, 24, 40]. These parameters are a critical determinant of post‐operative outcomes, particularly with the increasing shift toward patient‐specific alignment strategies in TKA. By integrating component sizing with alignment simulation and bone morphology analysis, such models offer a more comprehensive planning solution, minimizing complications related to malalignment or suboptimal fit [15].

Despite these promising advancements, the generalizability of current AI‐based models remains a significant concern. Most existing models are trained on retrospective, single‐centre data sets with limited demographic diversity and standardized imaging protocols, which may not reflect the variation encountered in broader clinical practice. Variability in patient anatomy, implant systems and radiographic techniques can markedly influence model performance, limiting its applicability without further external validation. The lack of prospective clinical evaluation therefore raises uncertainty as to whether the reported predictive accuracy translates into meaningful improvements in patient outcomes, surgical decision‐making or workflow efficiency. Prospective, multi‐centre validation is therefore essential to confirm the clinical utility and reliability of these models.

These broader concerns are reflected by several limitations within this review. First, heterogeneity in data sources, model architecture and ground truth definitions limited the ability to make direct comparisons across studies. Another limitation is the influence of implant positioning on the definition of the optimal component size. In several of the included studies, ground truth was determined solely by the implanted prosthesis size or by preoperative planning records. However, implant size is not an isolated parameter, and it is inherently linked to alignment and positioning choices made intraoperatively. This interdependence complicates the determination of true prediction accuracy, as the same patient anatomy may reasonably accommodate more than one component size depending on the positioning strategy. Accounting for this interplay between sizing and positioning will require integrating both parameters into ground truth determination and prediction metrics. A practical standardized reference could define ground truth as the component size derived from validated 3D preoperative planning that incorporates defined alignment and positioning parameters and is independently confirmed by another experienced surgeon. This would ensure a reproducible anatomical‐functional benchmark for assessing predictive accuracy.

Second, the independent accuracy or robustness of individual algorithms, such as stochastic gradient boosting or support vector machine, was not evaluated. However, this was beyond the scope of the current analysis, which aimed to compare predictive performance across different planning modalities rather than perform a head‐to‐head assessment of individual algorithm designs.

Third, important clinical endpoints, such as long‐term patient‐reported outcomes and implant survivorship or complication rates, were not consistently reported and therefore could not be analyzed. Finally, computational efficiency was reported in only a minority of studies, which limited the ability to assess the practical feasibility of different AI planning systems in time‐sensitive clinical workflows.

Overall, these findings underscore the potential of AI‐based planning to enhance implant sizing in TKA planning. To support clinical adoption, future work should focus on multicenter validation, integration of alignment and implant‐fit parameters and assessment of clinical effectiveness in real‐world settings.

## CONCLUSION

AI‐based models using imaging, particularly CT and MRI, demonstrate high accuracy for preoperative TKA implant sizing. While demographic‐only models are less precise, they may enhance performance when combined with imaging in multimodal approaches. Based on current evidence, x‐ray‐based models provide the best trade‐off between accuracy and practicality, while CT‐based models remain superior in precision but are less feasible for widespread clinical use.

## AUTHOR CONTRIBUTIONS

*Conceptualization*: Michael T. Hirschmann and Randa Elsheikh. *Methodology*: Michael T. Hirschmann and Randa Elsheikh. *Writing—original draft preparation*: Randa Elsheikh. *Writing—review and editing*: Randa Elsheikh, George Mihai Avram, Zainab Aqeel Khan, Rolf Huegli and Andrej M. Nowakowski. *Supervision*: Michael T. Hirschmann. All authors have read and agreed to the published version of the manuscript.

## CONFLICT OF INTEREST STATEMENT

The authors declare no conflicts of interest.

## ETHICS STATEMENT

The ethics statement is not available.

## Supporting information

Supplementary Material KSSTA.

## Data Availability

No new data were generated in this systematic review. Data extracted and analyzed from the included studies are available from the corresponding author upon reasonable request.

## References

[1] Arora J , Sharma S , Blyth M . The role of pre‐operative templating in primary total knee replacement. Knee Surg Sports Traumatol Arthrosc. 2005;13:187–189. 15824932 10.1007/s00167-004-0533-5

[2] Berend ME , Ritter MA , Hyldahl HC , Meding JB , Redelman R . Implant migration and failure in total knee arthroplasty is related to body mass index and tibial component size. J Arthroplasty. 2008;23:104–109. 18722310 10.1016/j.arth.2008.05.020

[3] Bertolino L , Ranzini MBM , Favaro A , Bardi E , Ronzoni FL , Bonanzinga T . Use of artificial intelligence on imaging and preoperatory planning of the knee joint: a scoping review. Medicina. 2025;61:737. 40283028 10.3390/medicina61040737 PMC12028754

[4] Bruns J , Kampen J , Kahrs J , Plitz W . Autogener meniskusersatz mittels rippenperichondrium: experimentelle ergebnisse. Orthopade. 2000;29:145–150. 10743636 10.1007/s001320050023

[5] Burge TA , Jones GG , Jordan CM , Jeffers JRT , Myant CW . A computational tool for automatic selection of total knee replacement implant size using X‐ray images. Front Bioeng Biotechnol. 2022;10:971096. 36246387 10.3389/fbioe.2022.971096 PMC9557045

[6] Chandrashekar AS , Suh Y , Fox JA , Mika AP , Moyer DC , Polkowski GG , et al. Development of a machine learning model for determining alignment in knees following total knee arthroplasty. J Arthroplasty. 2026;46(1):96–102. 10.1016/j.arth.2025.06.016 40499744

[7] Cichos KH , Hyde ZB , Mabry SE , Ghanem ES , Brabston EW , Hayes LW , et al. Optimization of orthopedic surgical instrument trays: lean principles to reduce fixed operating room expenses. J Arthroplasty. 2019;34:2834–2840. 31473059 10.1016/j.arth.2019.07.040

[8] Cucchi D , Menon A , Compagnoni R , Ferrua P , Fossati C , Randelli P . Significant differences between manufacturer and surgeon in the accuracy of final component size prediction with CT‐based patient‐specific instrumentation for total knee arthroplasty. Knee Surg Sports Traumatol Arthrosc. 2018;26:3317–3324. 29453487 10.1007/s00167-018-4876-8

[9] Dimri GP , Sharma GK , Mudholkar VM . Fabrication of deep curvature meniscus lenses. Appl Opt. 1989;28:17. 20548411 10.1364/AO.28.000017

[10] Gandhi RR , Manzotti A , Confalonieri N , Cerveri P . Comparison of CT‐based patient‐specific templating and digital radiography templating in total knee arthroplasty. J Arthrosc Joint Surg. 2016;3:17–21.

[11] Heller MT , Maderbacher G , Schuster MF , Forchhammer L , Scharf M , Renkawitz T , et al. Comparison of an AI‐driven planning tool and manual radiographic measurements in total knee arthroplasty. Comput Struct Biotechnol J. 2025;28:148–155. 40276217 10.1016/j.csbj.2025.04.009 PMC12019206

[12] Hernandez‐Vaquero D . Reliability of preoperative measurement with standardized templating in total knee arthroplasty. World J Orthop. 2013;4:287–290. 24147264 10.5312/wjo.v4.i4.287 PMC3801248

[13] Hinterwimmer F , Lazic I , Suren C , Hirschmann MT , Pohlig F , Rueckert D , et al. Machine learning in knee arthroplasty: specific data are key—a systematic review. Knee Surg Sports Traumatol Arthrosc. 2022;30:376–388. 35006281 10.1007/s00167-021-06848-6 PMC8866371

[14] Hiraoka A , Swinnen B , Vandeputte A , Franssen W , Leirs G . Optimizing operating room efficiency in robotic‐assisted total knee arthroplasty through manufacturing efficiency principles. J Exp Orthop. 2025;12:e70283. 40421405 10.1002/jeo2.70283 PMC12104826

[15] Hirschmann MT , von Eisenhart‐Rothe R , Graichen H , Zaffagnini S . AI may enable robots to make a clinical impact in total knee arthroplasty, where navigation has not!. J Exp Orthop. 2024;11:e70061. 39429889 10.1002/jeo2.70061 PMC11489858

[16] Hoenders CSM , Harmsen MC , van Luyn MJA . The local inflammatory environment and microorganisms in “aseptic” loosening of hip prostheses. J Biomed Mater Res Part B Appl Biomater. 2008;86:291–301. 10.1002/jbm.b.30992 18098200

[17] Katragadda S , Souza KD . Machine learning‐based automatic implant size prediction from CT images in total knee arthroplasty. Paper presented at: 2024 10th IEEE RAS/EMBS international conference for biomedical robotics and biomechatronics (BioRob). 2024: 1–4.

[18] Kobayashi A , Ishii Y , Takeda M , Noguchi H , Higuchi H , Toyabe S . Comparison of analog 2D and digital 3D preoperative templating for predicting implant size in total knee arthroplasty. Comput Aided Surg. 2012;17:96–101. 22309295 10.3109/10929088.2011.651488

[19] Kunze KN , Polce EM , Patel A , Courtney PM , Levine BR . Validation and performance of a machine‐learning derived prediction guide for total knee arthroplasty component sizing. Arch Orthop Trauma Surg. 2021;141:2235–2244. 34255175 10.1007/s00402-021-04041-5

[20] Kunze KN , Polce EM , Patel A , Courtney PM , Sporer SM , Levine BR . Machine learning algorithms predict within one size of the final implant ultimately used in total knee arthroplasty with good‐to‐excellent accuracy. Knee Surg Sports Traumatol Arthrosc. 2022;30:2565–2572. 35024899 10.1007/s00167-022-06866-y

[21] Kunze KN , Williams RJ , Ranawat AS , Pearle AD , Kelly BT , Karlsson J , et al. Artificial intelligence (AI) and large data registries: understanding the advantages and limitations of contemporary data sets for use in AI research. Knee Surg Sports Traumatol Arthrosc. 2024;32:13–18. 38226678 10.1002/ksa.12018

[22] Lambrechts A , Van Dijck C , Wirix‐Speetjens R , Vander Sloten J , Maes F , Van Huffel S . Preoperative prediction of optimal femoral implant size by regularized regression on 3D femoral bone shape. Appl Sci. 2023;13(7):4344.

[23] Lambrechts A , Wirix‐Speetjens R , Maes F , Van Huffel S . Artificial intelligence based patient‐specific preoperative planning algorithm for total knee arthroplasty. Front Robot AI. 2022;9:840282. 35350703 10.3389/frobt.2022.840282 PMC8957999

[24] Lan Q , Li S , Zhang J , Guo H , Yan L , Tang F . Reliable prediction of implant size and axial alignment in AI‐based 3D preoperative planning for total knee arthroplasty. Sci Rep. 2024;14:16971. 39043748 10.1038/s41598-024-67276-3 PMC11266554

[25] Lee OS , Raheman F , Jaiswal P . The accuracy of digital templating in the preoperative planning of total knee arthroplasties: a systematic review and meta‐analysis. Knee. 2024;47:139–150. 38394993 10.1016/j.knee.2024.01.006

[26] León‐Muñoz VJ , Lisón‐Almagro AJ , López‐López M . Planning on CT‐based 3D virtual models can accurately predict the component size for total knee arthroplasty. J Knee Surg. 2020;33:1128–1131. 31269525 10.1055/s-0039-1692645

[27] Li S , Liu X , Chen X , Xu H , Zhang Y , Qian W . Development and validation of an artificial intelligence preoperative planning and patient‐specific instrumentation system for total knee arthroplasty. Bioengineering. 2023;10(12):1417. 38136008 10.3390/bioengineering10121417 PMC10740483

[28] Marchand KB , Salem HS , Mathew KK , Harwin SF , Mont MA , Marchand RC . The accuracy of computed tomography‐based, three‐dimensional implant planning in robotic‐assisted total knee arthroplasty. J Knee Surg. 2021;35:1587–1594. 33932948 10.1055/s-0041-1729548

[29] Marino D , Patel J , Popovich Jr, JM , Cochran J . Patient demographics and anthropometric measurements predict tibial and femoral component sizing in total knee arthroplasty. Arthroplast Today. 2020;6:860–865. 33163600 10.1016/j.artd.2020.09.013 PMC7606840

[30] Miller AG , Purtill JJ . Accuracy of digital templating in total knee arthroplasty. Am J Orthop. 2012;41:510–512. 23431515

[31] Min M , Wang X , Urba R , Zhang W , Gao J , Fan L . Comparison of traditional template measurements and artificial intelligence preoperative planning in total knee arthroplasty. Front Surg. 2025;12:1573148. 40303951 10.3389/fsurg.2025.1573148 PMC12037627

[32] Oeding JF , Williams 3rd, RJ , Camp CL , Sanchez‐Sotelo J , Kelly BT , Nawabi DH , et al. A practical guide to the development and deployment of deep learning models for the orthopedic surgeon: part II. Knee Surg Sports Traumatol Arthrosc. 2023;31:1635–1643. 36773057 10.1007/s00167-023-07338-7

[33] Oettl FC , Oeding JF , Feldt R , Ley C , Hirschmann MT , Samuelsson K . The artificial intelligence advantage: supercharging exploratory data analysis. Knee Surg Sports Traumatol Arthrosc. 2024;32:3039–3042. 39082872 10.1002/ksa.12389

[34] Oettl FC , Zsidai B , Oeding JF , Hirschmann MT , Feldt R , Fendrich D , et al. Artificial intelligence‐assisted analysis of musculoskeletal imaging—a narrative review of the current state of machine learning models. Knee Surg Sports Traumatol Arthrosc. 2025;33:3032–3038. 40450562 10.1002/ksa.12702 PMC12310083

[35] Page MJ , McKenzie JE , Bossuyt PM , Boutron I , Hoffmann TC , Mulrow CD , et al. The PRISMA 2020 statement: an updated guideline for reporting systematic reviews. BMJ. 2021;372:n71. 33782057 10.1136/bmj.n71 PMC8005924

[36] Park J , Kim SE , Kim B , Lee S , Lee J‐J , Ro DH . A deep learning based automatic two‐dimensional digital templating model for total knee arthroplasty. Knee Surg Relat Res. 2024;36:38. 39605104 10.1186/s43019-024-00240-7 PMC11600925

[37] Park K‐B , Kim M‐S , Yoon D‐K , Jeon YD . Clinical validation of a deep learning‐based approach for preoperative decision‐making in implant size for total knee arthroplasty. J Orthop Surg. 2024;19:637. 10.1186/s13018-024-05128-6 PMC11463000 39380122

[38] Peek AC , Bloch B , Auld J . How useful is templating for total knee replacement component sizing? Knee. 2012;19:266–269. 21561779 10.1016/j.knee.2011.03.010

[39] Pietrzak JRT , Rowan FE , Kayani B , Donaldson MJ , Huq SS , Haddad FS . Preoperative CT‐based three‐dimensional templating in robot‐assisted total knee arthroplasty more accurately predicts implant sizes than two‐dimensional templating. J Knee Surg. 2019;32:642–648. 30068010 10.1055/s-0038-1666829

[40] Polce EM , Kunze KN , Paul KM , Levine BR . Machine learning predicts femoral and tibial implant size mismatch for total knee arthroplasty. Arthroplast Today. 2021;8:268–277.e2. 34095403 10.1016/j.artd.2021.01.006 PMC8167319

[41] Riechelmann F , Lettner H , Mayr R , Tandogan R , Dammerer D , Liebensteiner M . Imprecise prediction of implant sizes with preoperative 2D digital templating in total knee arthroplasty. Arch Orthop Trauma Surg. 2023;143:4705–4711. 36648539 10.1007/s00402-023-04772-7 PMC10374828

[42] Salzmann M , Hassan Tarek H , Prill R , Becker R , Schreyer AG , Hable R , et al. Artificial intelligence‐based assessment of leg axis parameters shows excellent agreement with human raters: a systematic review and meta‐analysis. Knee Surg Sports Traumatol Arthrosc. 2025;33:177–190. 39033340 10.1002/ksa.12362 PMC11716349

[43] Sershon RA , Courtney PM , Rosenthal BD , Sporer SM , Levine BR . Can demographic variables accurately predict component sizing in primary total knee arthroplasty? J Arthroplasty. 2017;32:3004–3008. 28583760 10.1016/j.arth.2017.05.007

[44] Simsek ME , Akkaya M , Gursoy S , Isik C , Zahar A , Tarabichi S , et al. Posterolateral overhang affects patient quality of life after total knee arthroplasty. Arch Orthop Trauma Surg. 2018;138:409–418. 29177951 10.1007/s00402-017-2850-4

[45] Sotozawa M , Kumagai K , Yamada S , Nejima S , Inaba Y . Patient‐specific instrumentation for total knee arthroplasty improves reproducibility in the planned rotational positioning of the tibial component. J Orthop Surg. 2022;17:403. 10.1186/s13018-022-03298-9 PMC9446751 36064582

[46] Tang A , Yeroushalmi D , Zak S , Lygrisse K , Schwarzkopf R , Meftah M . The effect of implant size difference on patient outcomes and failure after bilateral simultaneous total knee arthroplasty. J Orthop. 2020;22:282–287. 32581460 10.1016/j.jor.2020.06.009 PMC7305357

[47] Trickett RW , Hodgson P , Forster MC , Robertson A . The reliability and accuracy of digital templating in total knee replacement. J Bone Joint Surg Br. 2009;91–B:903–906. 10.1302/0301-620X.91B7.21476 19567854

[48] Wallace SJ , Murphy MP , Schiffman CJ , Hopkinson WJ , Brown NM . Demographic data is more predictive of component size than digital radiographic templating in total knee arthroplasty. Knee Surg Relat Res. 2020;32:63. 33225974 10.1186/s43019-020-00075-y PMC7682037

[49] Wolff RF , Moons KGM , Riley RD , Whiting PF , Westwood M , Collins GS , et al. PROBAST: a tool to assess the risk of bias and applicability of prediction model studies. Ann Intern Med. 2019;170:51–58. 30596875 10.7326/M18-1376

[50] Yu Y , Cho YJ , Park S , Kim YH , Goh TS . Development of an artificial intelligence model for predicting implant size in total knee arthroplasty using simple X‐ray images. J Orthop Surg. 2024;19:516. 10.1186/s13018-024-05013-2 PMC11348740 39192371

[51] Yue Y , Gao Q , Zhao M , Li D , Tian H . Prediction of knee prosthesis using patient gender and BMI with non‐marked x‐ray by deep learning. Front Surg. 2022;9:798761. 35360429 10.3389/fsurg.2022.798761 PMC8963922

[52] Yue Y , Wang X , Zhao M , Tian H , Cao Z , Gao Q , et al. Preoperative prediction of prosthetic size in total knee arthroplasty based on multimodal data and deep learning. In: 2019 IEEE 5th international conference on computer and communications (ICCC). Chengdu, China: IEEE; 2019, p. 2077–2081.

