# Supplementary material for: 3D imaging‐based AI models outperform demographic models and excel in tibial sizing compared with 2D models in total knee arthroplasty planning: A systematic review
Source: Knee Surg Sports Traumatol Arthrosc. 2026 Jan 8;34(3):1157–69. doi: 10.1002/ksa.70262 (PMC12948347; doi:10.1002/ksa.70262)
Supplement: Supplementary file 1 — Supplementary Material KSSTA. [file KSA-34-1157-s001.docx]

**Supplementary Table 1.** Detailed search strategy applied for study identification (from database inception to March 12, 2025).

| **Search terms** |
| --- |
| **PubMed** |
| **Concept 1: Artificial Intelligence**  "artificial intelligence"[tw] OR AI[tw] "machine learning"[tw] OR ML[tw] OR "deep learning"[tw] OR DL[tw] OR “neural network”[tw] OR computational[tw]  **Concept 2: TKA\TKR**  "total knee replacement"[tw] OR "total knee arthroplasty"[tw] OR "knee replacement"[tw] OR "Knee arthroplasty”[tw] OR TKR[tw] OR TKA[tw] OR "arthroplasty, replacement, knee"[majr] OR "arthroplasty, replacement, knee/methods"[majr] OR "knee joint/surgery"[mesh] OR "Knee Prosthesis"[mesh] OR "Knee Prosthesis"[majr] OR "arthroplasty, replacement, knee"[majr] OR "Knee Joint"[mesh]  **Concept 3: Implant size**  planning[tw] OR "component size"[tw] OR "implant size"[tw] OR “implant sizing”[tw] OR “component sizing”[tw] OR templating[tw] OR size[tw]  **Concept 1,2 and 3 (combined)**  ("artificial intelligence"[tw] OR AI[tw] "machine learning"[tw] OR ML[tw] OR "deep learning"[tw] OR DL[tw] OR “neural network”[tw] OR computational[tw]) AND ("total knee replacement"[tw] OR "total knee arthroplasty"[tw] OR "knee replacement"[tw] OR "Knee arthroplasty”[tw] OR TKR[tw] OR TKA[tw] OR "arthroplasty, replacement, knee"[majr] OR "arthroplasty, replacement, knee/methods"[majr] OR "knee joint/surgery"[mesh] OR "Knee Prosthesis"[mesh] OR "Knee Prosthesis"[majr] OR "arthroplasty, replacement, knee"[majr] OR "Knee Joint"[mesh]) AND (planning[tw] OR "component size"[tw] OR "implant size"[tw] OR “implant sizing”[tw] OR “component sizing”[tw] OR templating[tw] OR size[tw]) |
| **Embase**  1. artificial intelligence.mp or exp artificial intelligence/  2. machine learning.mp. or machine learning/  3. deep learning.mp or deep learning/  4. exp artificial neural network/ or neural network.mp.  5. computational.mp.  6. exp total knee arthroplasty/  7. total knee replacement.mp. and total knee arthroplasty/  8. component size.mp.  9. implant size.mp.  10. implant sizing.mp.  11. templating.mp.  12. size.mp. or size/  13. 1 or 2 or 3 or 4 or 5  14. 6 or 7  15. 8 or 9 or 10 or 11 or 12   1. 13 and 14 and 15 |
| **Scopus** (TITLE-ABS-KEY ("Total knee replacement") OR  TITLE-ABS-KEY ("Total knee arthroplasty")  OR  TITLE-ABS-KEY (TKA)  OR  TITLE-ABS-KEY (TKR))  AND  (TITLE-ABS-KEY ("artificial intelligence")  OR  TITLE-ABS-KEY ("machine learning") OR  TITLE-ABS-KEY (AI) OR  TITLE-ABS-KEY (ML) OR  TITLE-ABS-KEY ("deep learning") OR  TITLE-ABS-KEY (DL) OR  TITLE-ABS-KEY ("neural network") OR  TITLE-ABS-KEY (computational)  AND  (TITLE-ABS-KEY ( "implant size")  OR  TITLE-ABS-KEY ( planning) OR  TITLE-ABS-KEY (“implant sizing”) OR  TITLE-ABS-KEY (“component size”) OR  TITLE-ABS-KEY (templating) OR  TITLE-ABS-KEY (size)) |
| Cochrane Central Register(TITLE-ABS-KEY ("Total knee replacement") OR  TITLE-ABS-KEY ("Total knee arthroplasty")  OR  TITLE-ABS-KEY (TKA)  OR  TITLE-ABS-KEY (TKR))  AND  (TITLE-ABS-KEY ("artificial intelligence")  OR  TITLE-ABS-KEY ("machine learning") OR  TITLE-ABS-KEY (AI) OR  TITLE-ABS-KEY (ML) OR  TITLE-ABS-KEY ("deep learning") OR  TITLE-ABS-KEY (DL) OR  TITLE-ABS-KEY ("neural network") OR  TITLE-ABS-KEY (computational)  AND  (TITLE-ABS-KEY ( "implant size")  OR  TITLE-ABS-KEY ( planning) OR  TITLE-ABS-KEY (“implant sizing”) OR  TITLE-ABS-KEY (“component size”) OR  TITLE-ABS-KEY (templating) OR  TITLE-ABS-KEY (size)) |

**
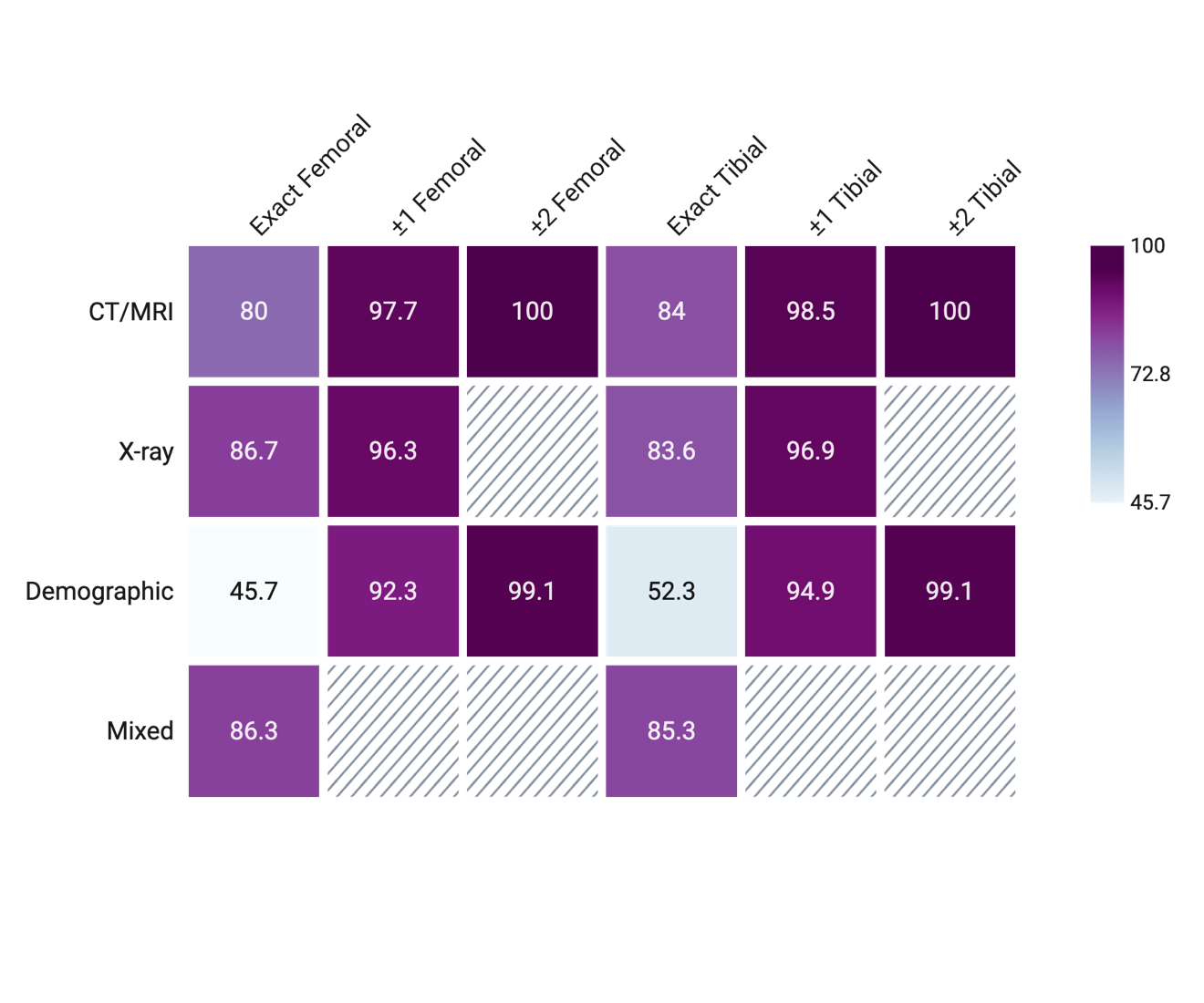
**

**Supplementary Figure 1.** Exact, ±1 size, and ±2 size accuracy of AI-based models utilizing CT/MRI, X-ray, demographic data, and mixed inputs (radiographs and demographic data).
